# Phosphate and potash solubilizing bacteria from Moroccan phosphate mine showing antagonism to bacterial canker agent and inducing effective tomato growth promotion

**DOI:** 10.3389/fpls.2023.970382

**Published:** 2023-03-08

**Authors:** B. Bouizgarne, M. Bakki, A. Boutasknit, B. Banane, H. El Ouarrat, S. Ait El Maalem, A. Amenzou, A. Ghousmi, A. Meddich

**Affiliations:** ^1^ Laboratory of Plant Biotechnology, Department of Biology, Faculty of Sciences, Ibn Zohr University (UIZ), Agadir, Morocco; ^2^ Laboratory of Agro-Food, Biotechnologies & Valorization of Plant Bioresources (AGROBIOVAL), Department of Biology, Faculty of Science Semlalia, Cadi Ayyad University (UCA), Marrakesh, Morocco

**Keywords:** biofertilizer, *Clavibacter michiganensis* subsp. *michiganensis*, *Pseudomonas*, PGPR, phosphate and potash solubilization, physiology, tomato growth and nutrition, yield

## Abstract

Most agricultural soils are facing limited phosphorus availability that challenges modern agriculture. Phosphate solubilizing microbia (PSM) has been explored extensively as potential biofertilizers for plant growth and nutrition, and harnessing phosphate rich areas could provide such beneficial microorganisms. Isolation of PSM from Moroccan rock phosphate led to the selection of two bacterial isolates, Bg22c and Bg32c, showing high solubilization potential. The two isolates were also tested for other *in vitro* PGPR effects and compared to a non-phosphate solubilizing bacterium Bg15d. In addition to phosphates, Bg22c and Bg32c were able to solubilize insoluble potassium and zinc forms (P, K, and Zn solubilizers) and produce indole-acetic acid (IAA). Mechanisms of solubilization involved production of organic acids as demonstrated by HPLC. *In vitro*, the isolates Bg22c and Bg15d were able to antagonize the phytopathogenic bacteria *Clavibacter michiganensis* subsp. *michiganensis*, causal agent of tomato bacterial canker disease. Phenotypic and molecular identification by 16S rDNA sequencing demonstrated delineation of Bg32c and Bg15d as members of the genus *Pseudomonas* and Bg22c as member of the genus *Serratia*. The two isolates Bg22c and Bg32c were further tested either alone or in a consortium and compared to the non-P, K, and Zn solubilizing *Pseudomonas* strain Bg15d for their efficacy to promote tomato growth and yield. They were also compared to treatment with a conventional NPK fertilizer. Under greenhouse conditions, *Pseudomonas* strain Bg32c remarkably improved the growth of whole plant height, root length, shoot and root weight, number of leaves and fruits, as well as fruit fresh weight. This strain also induced stomatal conductance enhancement. The strain also improved total soluble phenolic compounds, total sugars, protein, phosphorus and phenolic compounds contents compared to the negative control. All increases were more pronounced in plants inoculated with strain Bg32c in comparison with control and strain Bg15d. The strain Bg32c could be considered a potential candidate for formulation of a biofertilizer in order to improve tomato growth.

## Introduction

The production of healthy crops for the growing world population depends on plant growth and nutrition. After Nitrogen, phosphorus is the second most important nutrient for plant growth as it represents a key component of DNA, cell membranes, and cellular energy transport (Bouizgarne, 2022). Soil-available phosphorus exists in various organic and inorganic forms. However, most soils are poor in soluble, available phosphorus for plants. Indeed, although the content of phosphorus in soil is 0.05% (w/w), only 0.1% of it is available to plants, as the majority is represented by insoluble forms (Zhu et al., 2012). Thus, phosphorus (P) deficiency is considered a great challenge for modern agriculture as most agricultural soils are deficient in the nutrient. Nowadays, for optimal crop yields, conventional agriculture intensively uses chemical fertilizers. However, application of chemical fertilizers have two drawbacks: i) chemical fertilizers lead to freshwater and marine ecosystems pollution, and ii) plants benefit from only 20% of the applied chemical phosphorus fertilizers (Plaxton and Tran, 2011) and the remaining phosphate residue either accumulates in soils as insoluble mineral complex or is leached (Wakelin et al., 2004).

The most important source of phosphorous fertilizer is rock phosphate; it is therefore worthwhile to develop ecofriendly techniques by direct application of rock phosphate (Rajan et al., 1996) and or by using bacterial inoculi able to solubilize/mobilize rock phosphate from phosphate mines (Azaroual et al., 2020; Bouizgarne, 2022). Phosphate solubilizing microbia (PSM) with beneficial effects could be screened and directly used as biofertilizers. They could also be combined with chemical fertilizers (Adesemoye et al., 2009; Tahir et al., 2018). In general, these bacteria could convert insoluble phosphates into soluble forms through production of organic acids, cation chelation, or exchange reactions (Chung et al., 2005; Gulati et al., 2010). Furthermore, most phosphate solubilizing bacteria could also display other beneficial plant growth-promoting traits such as nitrogen fixation or phytohormone production (Bouizgarne, 2013; Wei et al., 2018). Currently, effects of phosphate solubilizing bacteria belonging to *Pseudomonas*, *Bacillus*, and actinomycetes have been described (Bouizgarne, 2013; Sharma et al., 2013; Bouizgarne and Ait Ben Aoumar, 2014; de Sousa et al., 2021; Bouizgarne, 2022).

The objective of the present study is to select soil phosphate solubilizing *Pseudomonads* from a Moroccan phosphate mine, to assess their *in vitro* growth-promoting abilities and antagonism against tomato bacterial canker agent (*Clavibacter michiganensis* subsp. *michiganensis)*, as well as their beneficial effects on the growth of cherry tomato in greenhouse conditions. To our knowledge, this is the first report on the isolation of phosphate solubilizing fluorescent *Pseudomonas* from a Moroccan phosphate mine with plant growth-promotion abilities.

## Materials and methods

### Isolation and screening for phosphate solubilizing bacteria

Bacterial strains were isolated from soil samples collected in bulk from a phosphate mine. Soil dilutions were performed, and 0.1 mL of each dilution was spread on King B medium. After incubation at 28°C for 48 h, bacteria showing fluorescence under UV at 365 nm were Gram stained and checked for cytochrome-C oxidase (oxidase test). The capacity of the isolates to solubilize inorganic phosphate was tested on National Botanical Research Institute’s Phosphate growth (NBRIP) medium (per liter: 10 g glucose, 0.1 g (NH_4_)_2_SO4, 0.2 g KCl, 0.25 g MgSO_4_.7H_2_O, 5 g MgCl_2_. 6H_2_O, and 5g tri-calcium phosphate (TCP) (Nautiyal, 1999) and solubilization index (PSI) was calculated according to the following formula:


PSI = [Diameter of halo zone+Colony diameter]/Colony diameter

For quantitative estimation of phosphate solubilization in liquid NBRIP medium, 20 mL of NBRIP containing TCP were inoculated with 100 μL of standardized bacterial suspensions at a final concentration of 10^6^ CFU.mL^-1^, obtained from a 24 hour preculture in King B (KB) medium. The flasks were incubated at 28°C in the dark under shaking at 180 rpm. After 48 h, the phosphate solubilization was monitored by colorimetry in culture supernatant at 600 nm, using a UV-visible spectrophotometer (UV- 3100PC, VWR), according to the Fiske and Subbarow (1925) method and expressed as mg. L^-1^ released P. A calibration curve was recorded using KH_2_PO_4_ and the pH variation was estimated by recording pH at starting conditions and after 48h.

High-performance liquid chromatography (HPLC) (Ultimate 3000 Dionex) coupled with a Mass spectrometry (Exactive Plus de Thermo Scientific) equipped with a BDS Hypersil C18 (150 x 4.6) mm x 5µm) was used to identify and quantify organic acids. Samples were collected, filtered through a 0.22 um cellulose membrane, and injected into the chromatographic column. The mobile phase was 0.1% H_3_PO_4_ (pH 1.81) with a 0.5 mL min^-1^ flow rate and a 100 µL injection per sample according to Marra et al. (2015). The acquisition time of the chromatograms was estimated to be 30 min with 30 min intervals between runs. Detection was performed by UV at 210 nm with a diode array detector (DAD).

Detection of organic acids was performed using the following organic acids (Supelco/Sigma Aldrich) as analytical standards with typical retention times: 2-keto gluconic acid (2.10 min), phytic acid (3.2 min), gluconic acid (3.74 min), tartaric acid (4.02 min), quinic acid (4.03 min), oxalic acid (4.5 min), shikimic acid (4.81min), iso-citric acid (4.98 min), malonic acid (5.23 min), ascorbic acid (5.4 min), citric acid (7.61 min), fumaric acid (8.09 min), succinic acid (8.86 min), propionic acid (9 min), maleic acid (9.27 min), and benzoic acid (12.5 min).

### Potassium solubilization

Potassium solubilization by the isolates was carried out on Aleksandrov agar medium (Etesami et al., 2017), containing 5.0 g/L glucose, 0.5 g/L MgSO_4_. 7H_2_O, 0.1 g/L CaCO_3_, 0.006 g/L FeCl_3_, 2.0 g/L Na_2_ HPO_4_, and feldspath (2.0 g/L) as sole K source. The plates were then incubated for 2 days at 28°C. Potassium solubilization index (KSI) was calculated as for phosphate.

### Zinc solubilization

The zinc solubilization was investigated in Bunt and Rovira (B & R) medium (Bunt and Rovira, 1955) (glucose: 20.0g, peptone: 1.0g, yeast extract: 1.0g, (NH_4_)_2_ SO_4_: 0.50g, K_2_ HPO_4_: 0.40g, MgCl_2_: 0.10g, FeCl_3_: 0.01g and distilled water: 1000 mL, pH 6.7) containing 0.1% insoluble zinc oxide. Fresh cultures of bacteria were spot inoculated at the center of the plate in triplicates with toothpicks and incubated for 48 hours in the dark at 26°C. Appearance of halo zones around colonies reflected Zn solubilization. Halo zone diameter around each colony and the colony diameter were measured. Zn solubilization index (ZSI) was calculated as for phosphate.

### Indole-3-acetic-acid production

Production of IAA was demonstrated according to Bric et al. (1991). Isolates were incubated in Luria Bartani (LB) medium at 28°C for 2 days and positive colonies were revealed by Salkowski reagent. For quantitative measurement, liquid LB medium containing 0.1 g/L of L-Trp was seeded with isolates and incubated for 3 days at 28°C. IAA amounts were determined according to Gravel et al. (2007). A calibration curve was recorded using IAA (Sigma-Aldrich).

### Antagonism towards *Clavibacter michiganensis* subsp. *michiganensis*

The isolates were cultured on the King B agar at 28°C for 24 h. The antibacterial effects against *Clavibacter michiganensis* subsp. *michiganensis* was determined by the plate diffusion method in NBY agar medium using the agar cylinder method (Bouizgarne et al., 2006). Ten mm diameter cylinders were cut and placed on NBY agar medium freshly seeded with a bacterial suspension containing 10^6^ UFC/mL *Clavibacter michiganensis* subsp. *michiganensis.* Plates were allowed to pre-diffuse at 4°C for 4 h before incubation at 28°C. Three replicates were performed for each isolate and diameters of inhibition zones (20 mm or more) were measured after 24 h.

### Greenhouse experiments

Inocula of three selected isolates Bg22c, Bg32c, and Bg15d and the consortium of Bg22c and Bg32c were prepared by growing the bacteria in King B liquid medium at 28°C to an optical density of 1 at 600 nm (approximately 10^9^ CFU/mL). Cherry tomato seeds were surface disinfected with sodium hypochlorite for 10 min, rinsed five times with sterile distilled water, and allowed to germinate. Germination was performed in plastic Petri dishes containing sterile wet filter paper discs. The dishes were incubated for 5 days at 26°C in the dark. The germinated seeds were then transplanted into plastic trays containing sterilized peat. At four-leaf stage, the seedlings were transplanted into plastic pots containing 5 kg of soil sterilized at 180°C for 3 hours. The soil used had the following characteristics: sand 51%; clay 19%; silt 30%; organic matter 1%; total organic carbon 0.39%; available phosphorus 11 ppm nitrogen 0.84 mg.g^-1^; pH 8.6; and EC 0.19 mS.cm^-1^.

The experiment consisted of six treatments: (1) Control: plants receiving no biofertilizer, (2) Chemical fertilizer (CF): plants receiving the optimal dose of conventional NPK fertilizer, (3) Bg22c: plants receiving only Bg22c bacteria, (4) Bg32c: plants receiving only Bg32c bacteria, (5) Bg15d: plants receiving only Bg15d bacteria, and (6) Bg22c + Bg32c: plants receiving a combination of Bg22c and Bg32c. Each bacterial suspension or combination contained 10^6^ UFC/mL. The experiment was set up in a fully randomized design with eight replicates per treatment. After 15 days of transplantation, plant inoculations were performed by adding 5 mL of each of the three bacterial suspension or a suspension of the mixture of Bg32c and Bg22c to the vicinity of the plant roots. After 15 days of the first inoculation, a second inoculation was performed by placing an additional 4 mL of the bacterial suspensions to ensure infection of the newly formed roots. Seedlings were grown with a day/night cycle of 16/8 h and an average temperature of 25.5°C, average relative humidity of 68.5%, and light of 410 μm^-2^ s^-1^. The whole experiment was conducted over 120 days.

#### Effects on plant growth parameters

Tomato growth was assessed by measuring shoot and root length, number of leaves, number of fruits, shoot and root fresh and dry weight, and fruit weight. Shoots were cut and weighed to determine their fresh weight, while the root parts were carefully washed, gently dried to remove any free surface moisture, and weighed to determine the fresh weight of the roots. Shoot and root dry weights were evaluated after drying in an oven at 80°C for 72 h until a constant weight was obtained.

#### Stomatal conductance

Stomatal conductance was measured on the leaves of the second rank using a portable porometer (Decagon Devices, Inc., Pullman, WA, USA) between 10:00 am and 12:00, as described by Harley et al. (1992).

#### Photosynthetic pigments content and chlorophyll fluorescence

The determination of the concentration of chlorophyll pigments (chlorophyll a, b, total chlorophyll, and carotenoids) was performed according to the method described by Arnon (1949). Extraction of photosynthetic pigments was performed on frozen leaf samples using 80% cold acetone. After centrifugation at 10000g for 10 minutes, the absorbance of the supernatant was read at 480, 645, and 663 nm using a UV-visible spectrophotometer (spectrophotometer UV-3100PC, VWR).

Chlorophyll fluorescence (Fv/Fm) was measured by an OS30p fluorometer (Optisciences, USA) on the upper side of the second fully developed leaf from the apex under dark adaptation (obscuring for 30 min). Fv/Fm was measured by transmission at 650 nm on a leaf area of 12.5 mm^2^. The fluorescence signal was recorded for a second at an acquisition speed of 10 ms (Strasser and Strasser, 1995).

#### Phosphorus content

For phosphorus mineral analyses, dried samples of tomato leaves were ground with a coffee grinder. Leaves and fruit powder (0.5 g) were incinerated in a muffle furnace before acid extraction and phosphorus concentration was estimated by the Olsen method (Olsen and Sommers, 1982).

#### Total soluble sugars (TSS), phenolic compounds, and protein content

Total soluble sugars were determined in 0.1 g of the frozen leaf and fruit powder in 80% (v/v) ethanol. Total soluble sugars content was determined according to Dubois et al. (1956). Of the obtained supernatant, 0.2 mL was mixed with 0.2 mL of phenol and 1 mL of concentrated sulfuric acid. The content of TSS was determined by measuring the absorbance at 485 nm and calculated using the standard glucose curve. Frozen leaf and fruit (0.1 g) were homogenized in a cold mortar with 4 mL of 1 M phosphate buffer (pH 7) containing 5% polyvinylpolypyrrolidone. The homogenate was centrifuged at 18.000xg for 15 min at 4°C. Protein content was determined according to the technique described by Bradford (1976). The determination of phenolic compounds was carried out according to the method described by Macheix et al. (1990). The extraction was performed in roots by using methanol. 200 mg of roots were blended at 4°C in 2 ml 80% methanol. After stirring for 15 minutes and centrifugation at 7000 g for 3 min, the supernatant was used to measure total soluble phenolic compounds content. The estimation of the total phenols’ concentration (milligram equivalent of caffeic acid per gram fresh weight) was performed using the Folin Ciocalteu reagent. The reaction mixture consisted of 50 μl of phenolic extracts diluted in 2 mL distilled water and 0.5 ml Folin Ciocalteu reagent. After agitation, 0.5 ml of a solution of Na_2_CO_3_ (20%) was added and the mixture was incubated for 30 minutes at 40°C. Blanc was prepared by adding 0.5 mL Folin- Ciocalteu to 0.5 ml of 20% Na_2_CO_3._ The absorbance was measured at 760 nm. Phenolic determination of each sample was carried out in triplicate.

### Identification of the bacterial isolates

The characterization of isolates was performed by observing phenotypic traits including colony morphology, color, size, elevation, margin, Gram staining, and fluorescence under UV light (254 m). Further, catalase, oxidase, tween, and levan tests were assayed. Other traits, such as H_2_S production and the ability to hydrolyze gelatin and starch, were also performed by using API 20NE strips (Biomérieux-France). All tests were made in triplicate. Molecular characterization of the three isolates was performed using 16S rRNA gene sequences as follows: the selected bacteria were grown on King B broth on a rotary shaker (180 rpm) at 28°for 48h. After centrifugation, total DNA was extracted with the “MagPurix Bacterial DNA extraction kit” using the MagPurix extractor robot. Purified bacterial DNA was estimated by Nanodrop 8000. The bacterial 16S rRNA gene was amplified using the universal 16S rRNA primers FD1 (5’- AGAGTTTGATCCTGGCTCAG-3’) and RP2 (5’-TACGG CTACCTTGTTACGACTT- 3’). The PCR was carried out with a 25-µL final volume, containing 10 µL of PCR Master Mix (2X) (HS MyTaq DNA polymerase kit from Bioline), 1.5 µL of each primer, 130 ng of DNA, and purified water. PCR was performed using an ABI “Verity” thermal cycler (Applied Biosystems, Foster City, CA) and the following PCR Program: 95°C, 2 min; (95°C, 30 s; 52°C, 30 s; 72°C, 30 s) 35x; 72°C, 3 min. The final amplicon size was 1500bp. 8 μL of PCR products were deposited on 1% agarose gel in the presence of the 1 kb molecular weight ladder and the gel was visualized by the “G Box” documentation system. Sequencing was performed on almost complete 16S rDNA sequences (about 1500bp) gene using the 3130XL Dye Terminator Cycle Sequencing (DTCS) Quick Start kit (Applied Biosystems) according to manufacturer instructions. The thermocycling conditions for the cycle sequencing reaction were as follows: 25 cycles of 96°C for 1 min, 96°C for 10 s, 50°C for 5 s, and 60°C for 4 min, followed by a 4°C infinite hold. The Sephadex G50 superfine (Sigma Aldrich) was used to remove unincorporated dye terminators from the cycle sequencing reaction, according to manufacturer’s instructions, with an additional 300 μL wash of the column with distilled H_2_O and centrifugation at 1500×g for 3 min prior to applying the sample to the column. The sequencing reaction was performed in two directions using the forward and reverse primers FD1 and RP2 to cover the length of the 16S rRNA gene. Cleaned PCR products were used as a template for the cycle sequencing reaction (Berrada et al., 2012). Forward and reverse sequencing were performed using Big Dye Terminator version 3.1 cycle sequencing kit (Applied Biosystems, Foster City, CA) according to the manufacturer’s instructions. Sequence assembly was performed using the program Codoncode. After trimming, forward and reverse sequences were aligned and then assembled to obtain the full contiguous sequence. Then, the sequences were submitted to basic local alignment search tool (BLAST) provided online by the National Center for Biotechnology Information (NCBI, Bethesda, MD, USA). Sequences were deposited at Genbank under the following accession numbers: Bg32c (ON394488.1), Bg22c (ON776891.1), and Bg15d (ON385873.1). The nearest 16S rRNA gene sequences were downloaded. Clustal W was used for multiple alignment with available almost-complete sequences of type strains of the family Pseudomonadacea and the determination of phylogenic relationships was performed by the phylogenic analysis Mega 11 software (Stecher et al, 2020; Tamura et al., 2021). The tree was built using the neighbor-joining method (Saitou and Nei, 1987) with statistic method of Maximum Likelihood. Tree topology was evaluated by bootstrap analyses (1000 replicates) (Felsenstein, 1985). Isolates were regarded as belonging to a species when sequence similarity with the species type strain was at least 98.8%.

### Data analysis

Experimental data presented are mean values based on eight replicates ± standard error per treatment. Statistical analysis was performed with SPSS 23.0 software for Windows. An analysis of variance (ANOVA) was performed to determine the significance of differences in plant responses to the different treatments applied. Significant differences among means were determined by Tukey’s *post hoc* at p< 0.05. Graphs and Principal Component Analysis (PCA) were performed using Graphpad Prism 9 software.

## Results

### *In vitro* plant growth promotion (PGP) characteristics of the isolates

**Figure 1:**
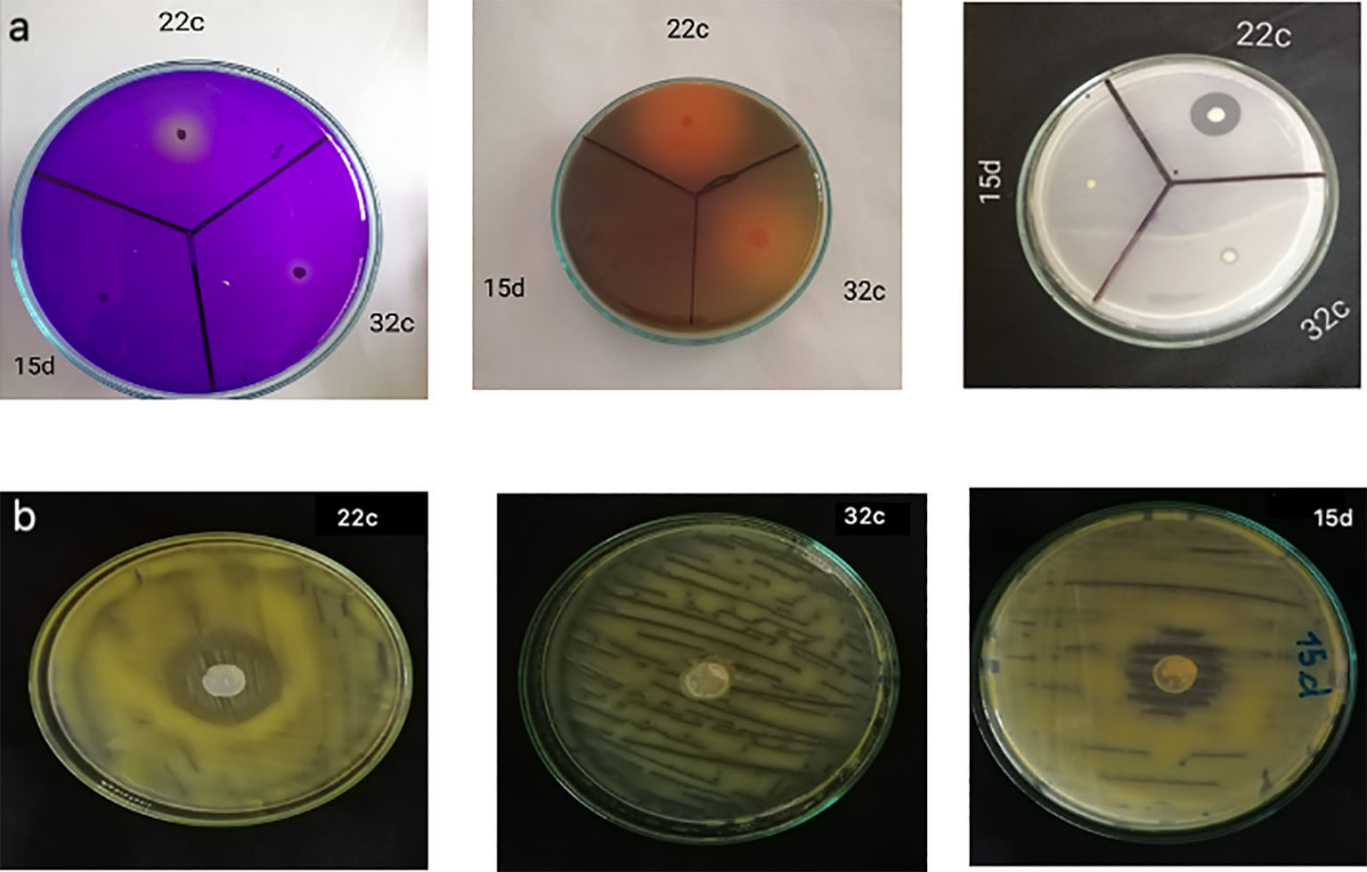
PGPR traits of the three bacterial isolates Bg22c, Bg32c and Bg15d,: **(A)** Solubilization of insoluble forms of phosphorus, potassium and zinc by the bacterial isolates, **(B)** antagonism against *C. michiganensis* subsp. *michiganensis*.

**Table 1** and **Figure 1** show the *in vitro* PGP traits of the three selected isolates. The isolates Bg22c and Bg32c showed strong phosphate solubilization in comparison to Bg15d. They also showed potassium and zinc solubilization while Bg15d did not. Phosphate solubilization index (PSI) was 3.83 and 2.85 for Bg22c and Bg32c, respectively. Potassium solubilization index (KSI) was 2.50 and 4.70 for Bg22c and Bg32c, respectively. Bg15d showed a poor P, K, and Zn solubilization potential. Phosphate solubilization was concomitant to a drop in pH values.

**Table 1:** *In vitro* PGPR characteristics of the phosphate solubilizing *Serratia* sp. Bg22c and *Pseudomonas* sp. Bg32c compared to non-P solubilizing *Pseudomonas* sp. strain Bg15d.

Isolate	PSI	KSI	ZSI	pH at 48 h	Production of IAA (ug. mL^-1^)	Antagonsism of *C.* michiganensis*
Control	not applicable	not applicable	not applicable	6.8 ± 0.01	negative	negative
Bg22c	3.83	2.50	3.5	4.73 ± 0.02	155.00 ± 1.83	2.75 ± 0.05
Bg32c	2.85	4.70	2.4	5.12 ± 0.03	90.62 ± 0.21	0.00 ± 0.00
Bg15d	0.01	0.01	0	6.29 ± 0.01	116.00 ± 0.25	2.9 ± 0.1

PSI, phosphate solubilization Index; KSI, potassium solubilization index; ZSI, zinc solubilization index; * Diameter of Inhibition Zone (cm).

The two isolates Bg22c and Bg32c brought the medium pH value down compared to control, while the pH value was not significantly changed for isolate Bg15d. The pH of the medium at initial conditions was 6.8 and dropped to 4.73 for Bg22c and 5.12 for Bg32c after 48 hours. The drop in pH value suggests involvement of organic acids produced in the culture medium. **Supplementary Figure 1** shows the main organic acids detected by HPLC in the supernatant of liquid NBRIP culture medium. The HPLC profiles of culture supernatants from the two isolates showed the presence of gluconic acid produced by Bg22c while Bg32c produced mainly oxalic acid. All the isolates were able to produce IAA *in vitro.* IAA amounts produced *in vitro* were 155, 90.62. and 116.00 respectively for Bg22c, Bg32c, and Bg15d. Bg22 and Bg15d showed inhibition of *Clavibacter michiganensis* subsp. *michiganensis in vitro* while Bg32c showed no inhibition. 

### Greenhouse experiments

#### Effects on shoot and root length and leaves number

**Table 2** and **Supplementary Figure 2** show the results of the effects of different applied treatments on shoot length (SL) and the number of leaves (NL) at the end of the experiment (120 days). The application of phosphate solubilizing bacteria significantly improved shoot height and number of leaves compared to the negative control. After 120 days of cultivation, the application of these bacteria, with the exception of Bg15d, showed no significant difference in SL compared to the positive control. Indeed, Bg22c, Bg32c, and their combination improved SL by 17.0, 19.2, and 18.6% respectively compared to the negative control. Similarly, number of leaves (NL) was improved in plants inoculated with Bg22c, Bg32c, and their combination by 17.6, 22.1, and 13.6% respectively compared to the negative control. The results in **Table 2** show the effects on root growth of cherry tomato. Tomato plants inoculated with strain Bg22c and Bg32c had a greater root length (RL) than the negative control plants by about 29 and 35% respectively. In addition, the application of strain Bg32c exhibited a greater root length than that obtained in tomato plants treated with NPK chemical fertilizers by about 5.4%.

**Table 2:** Growth chracteristics of cherry tomato plants after application of phosphate solubilizing *Serratia* sp. Bg22c and *Pseudomonas* sp. Bg32c and their consortium (Bg22c +Bg32c) compared to non-solubilizing isolate, *Pseudomonas* sp. Bg15d, and a chemical fertilizer (CF) on cherry tomato shoot and root length, number of leaves, shoot and root fresh weight, shoot and root dry weight, fruit fresh weight, and number of fruits.

	SL (cm)	RL (cm)	NL	SFW (g)	RFW (g)	SDW (g)	RDW (g)	FFW (g)	NF
Control	142.9 ± 3.6 ^c^	23.5 ± 1.8 ^b^	24.9 ± 2.0 ^d^	110.5 ± 6.7 ^b^	16.4 ± 0.4 ^b^	82.2 ± 2.2^b^	13.0 ± 0.5 ^c^	23.1 ± 1.7 ^b^	10.4 ± 1.8 ^c^
CF	176.5 ± 15.7 ^a^	30.1 ± 4.6 ^a^	32.9 ± 2.1 ^a^	126.5 ± 2.9 ^a^	18.4 ± 0.9 ^ab^	98.9 ± 2.1^a^	14.5 ± 0.7 ^ab^	31.8 ± 5.2 ^a^	22.0 ± 2.8 ^a^
Bg22c	167.1 ± 16.0 ^ab^	30.38 ± 3.6 ^a^	29.3 ± 3.5 ^abc^	118.3 ± 10.0 ^ab^	18.7 ± 1.0 ^ab^	94.3 ± 4.2 ^a^	13.9 ± 0.7 ^bc^	29.4 ± 1.7 ^a^	16.6 ± 2.3 ^b^
Bg32c	170.3 ± 8.1 ^ab^	31.75 ± 4.0 ^a^	30.4 ± 3.8 ^ab^	125.4 ± 3.5 ^a^	19.4 ± 1.7 ^a^	97.2 ± 2.0 ^a^	15.8 ± 1.0 ^a^	31.1 ± 2.9 ^a^	17.1 ± 1.2 ^b^
Bg15d	155.4 ± 5.5 ^bc^	29.38 ± 4.0 ^a^	25.9 ± 2.6 ^cd^	118.4 ± 5.1 ^ab^	17.5 ± 1.7 ^ab^	91.2 ± 3.7 ^a^	14.0 ± 0.7 ^bc^	28.0 ± 3.1 ^a^	12.0 ± 1.2 ^bc^
Bg22c +Bg32c	169.5 ± 11.2 ^ab^	28.88 ± 3.4 ^ab^	28.3 ± 2.8 ^bcd^	119.9 ± 2.9 ^a^	17.8 ± 1.8 ^ab^	95.7 ± 3.3 ^a^	14.6 ± 0.8 ^ab^	29.4 ± 4.3 ^a^	14.0 ± 2.1 ^bc^

Control, no fertilizers used; CF, chemical fertilizer (positive control), optimal dose of NPK; Bg22c, inoculation with *Serratia* sp, isolate Bg22c; Bg32c, with *Pseudomonas* sp, isolate Bg32c; Bg15d, with *Pseudomonas* sp, isolate Bg15d; and Bg22c +Bg32c, with the bacterial consortium of isolates Bg22c and Bg32c; SL, shoot length; RL, root length; NL, number of leaves; SFW, shoot fresh weight; RFW, root fresh weight; SDW, shoot dry weight; RDW, root dry weight; FFW, fruit fresh weight; NF, number of fruits, The values of each parameter labeled by different letters indicate significant differences assessed by Tukey’s test after performing a one-way ANOVA (P< 0,05).

#### Effects on shoot and root biomass and number of fruits

**Table 2** shows the effects of treatments on shoot and root fresh biomass (SFW and RFW) and dry biomass (SDW and RDW) compared to the negative control. Inoculation with strain Bg32c resulted in significant differences compared to the negative control. Highest enhancements in SFW and SDW were registered when the isolate Bg32c was applied either alone or in combination with the isolate Bg22. In addition, no significant differences were found between the effects of Bg32c application in comparison with the positive control. Enhancements by 13.5% and 18.4% were registered respectively in SFW and SDW when Bg32c was applied. Combination of Bg22c + Bg32c resulted in enhancement by 8.5% and 16.5% respectively in SFW and SDW, while application of the isolate Bg22c alone resulted only in enhancements by 7% and 14.8% respectively in SFW and SDW, and application of the isolate Bg15d alone resulted in enhancement by 7.1% and 11.0% respectively in SFW and SDW. Similarly, the largest enhancements in RFW and RDW were registered when isolate Bg32c was applied alone, for which significant differences were found compared to the negative control. Indeed, enhancements by 18.4% and 21% were registered respectively in RFW and RDW. Application of the isolate Bg22c resulted only in enhancements by 13.7% and 6.8% respectively in RFW and RDW, while combination of Bg22c and Bg32c resulted in enhancement of 8.2% and 12.3% respectively in RFW and RDW and application of the isolate Bg15d resulted in enhancement by 6.6% and 7.7% respectively in RFW and RDW.

Application of the phosphate solubilizing bacteria also caused significant increases in Cherry tomato fruit yield. All applied bacteria increased the fruit fresh weight (FFW) and fruit number (FN) of cherry tomato (**Table 2**). Strain Bg32c was the most effective inoculant compared to treatments with strain Bg22c, Bg15d, and the combination of Bg22c and Bg32c. Bg32c inoculated plants showed approximately 34.6 and 65.1% higher FFW and FN, respectively, compared to negative control plants.

#### Stomatal conductance and chlorophyll fluorescence

**Figure 2:**
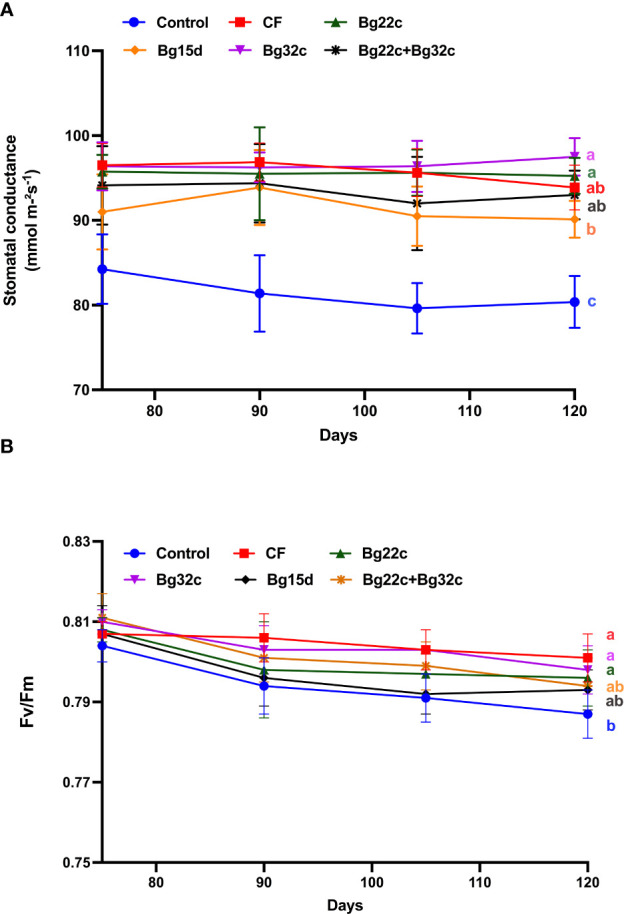
Effects of different phosphate solubilizing bacteria and chemical fertilizer (NPK) on stomatal conductance **(A)** and chlorophyll fluorescence (Fv/Fm) **(B)** in cherry tomato. Control^-^: absence of the tested biofertilizers, CF: chemical fertilizer (positive control): application of the optimal dose of NPK, Bg22c: inoculation with *Serratia* sp. isolate Bg22c, Bg32c: with *Pseudomonas* isolate Bg32c, Bg15d: with *Pseudomonas* isolate Bg15d, and Bg22c + Bg32c: with the bacterial consortium of isolates Bg22c and Bg32c. Different letters are significantly different (P<0.05) by Tukey test.

The application of PSB also resulted in an improvement of the physiological parameters of tomato compared to the negative control (**Figure 2**). Changes in stomatal conductance and chlorophyll fluorescence (Fv/Fm) of tomato during 120 days of culture showed a significant difference between plants inoculated with the bacteria and those not inoculated. Inoculation of tomato plants with Bg32c bacteria recorded an increase in stomatal conductance by 14.4, 18.3, 21.0, and 21.3% after 75, 90, 105, and 120 days of culture respectively compared to the negative control. Similarly, it can be seen that the application of Bg32c resulted in a significant improvement in Fv/Fm compared to the negative control by 2% throughout the culture period. However, the application of strain Bg22c, strain Bg32c, and their combination did not show significant improvement in stomatal conductance and chlorophyll fluorescence compared to NPK treatment.

#### Chlorophyll and carotenoid pigment content

**Figure 3:**
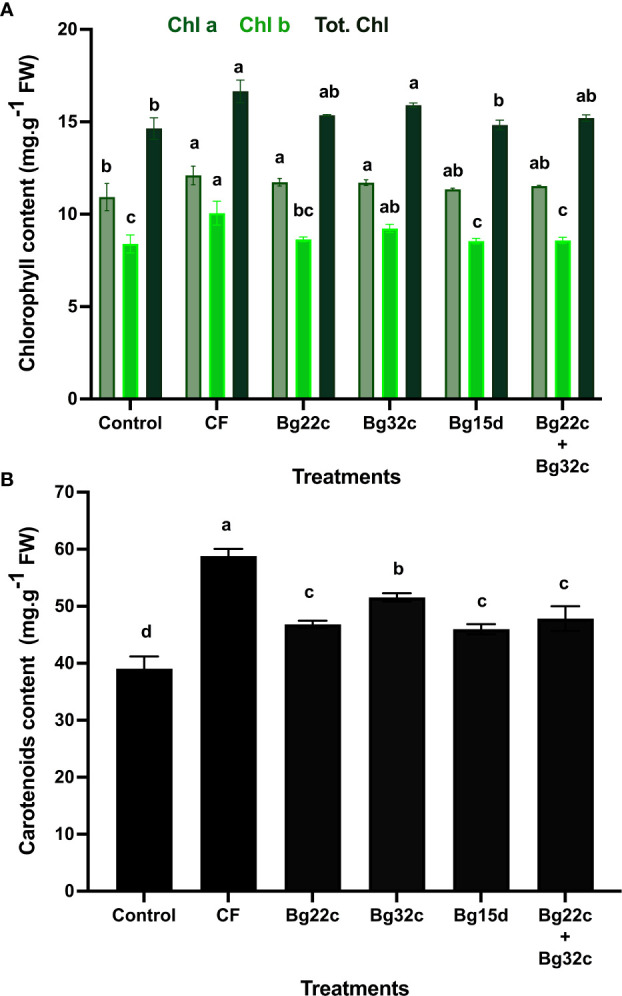
Effects of different phosphate solubilizing bacteria and chemical fertilizer (NPK) on chlorophyll **(A)** and carotenoid **(B)** pigment content in cherry tomato. Control: absence of the tested biofertilizers, CF: chemical fertilizer (positive control): application of the optimal dose of NPK, Bg22c: inoculation with *Serratia* sp. isolate Bg22c, Bg32c: with *Pseudomonas* sp. isolate Bg32c, Bg 15d: with *Pseudomonas* sp. strain Bg15d, and Bg22c + Bg32c: with the bacterial consortium of isolates Bg22c and Bg32c. Different letters are significantly different (P<0.05) by Tukey test.

We evaluated the protective role of the application of PSM on chlorophyll pigment content in cherry tomato. The results obtained revealed that the application of these bacteria caused a significant increase (p< 0.05) in the content of chlorophyll a (Chl a), chlorophyll b (Chl b), total chlorophyll (Chl T), and carotenoids (**Figure 3**). Indeed, inoculation of tomato plants with Bg32c significantly increased Chl a by 7.2%, Chl b by 10.0%, Chl T by 8.6, and carotenoids by 32.0% compared to untreated and uninoculated tomato plants. Inoculation of plants with strain Bg32c alone significantly improved chlorophyll pigment content compared to the other different bacteria applied.

#### Total soluble sugar, total soluble phenols, and protein content

The level of sugars and proteins in leaves and fruits after 120 days of growth was higher in plants inoculated with the bacteria and treated with the chemical fertilizer NPK than in the untreated ones (**Table 3**). The level of sugars in leaves and fruits was much higher in plants inoculated with Bg32c, with increases of 30% and 60% respectively compared to the negative control plants (**Table 3**). Similarly, the highest level of protein in fruits was found in plants inoculated with Bg32c and the combination followed by plants grown in NPK-amended soil with increases by 120.2, 70.9, and 41.2% respectively compared to the negative control (**Table 3**). The application of the three strains Bg22c, Bg32c and Bg15d resulted in an enhancement of phenolic compounds’ content in comparison with control. In addition, the consortium of Bg22c and Bg32c could also enhance phenols. Bg32c showed the maximum enhancement by up to 4.8-fold compared to the control.

**Table 3:** Effects of phosphate solubilizing bacterial isolates Bg22c and Bg32c and their consortium (Bg22c +Bg32c) compared to non-solubilizing isolate, *Pseudomonas* sp. Bg15d, and a chemical fertilizer (CF) on total soluble proteins, total sugars, and total soluble phenolics in cherry tomato plants.

		Control	CF	Bg22c	Bg32c	Bg15d	Bg22c+Bg32c
**Proteins** **(mg. g^-1^ FM)**	**Leaves**	**63.16 ± 0.49^b^**	**84.12 ± 1.15^a^**	**82.03 ± 0.32^a^**	**84.75 ± 0.64^a^**	**84.12 ± 1.47^a^**	**84.46 ± 0.98^a^**
	**Fruits**	**107.54 ± 1.96^e^**	**151.88 ± 3.95^c^**	**183.77 ± 4.57^b^**	**237.10 ± 12.42^a^**	**149.86 ± 4.16^c^**	**133.91 ± 4.16^d^**
**Sugars** **(mg. g^-1^ FM)**	**Leaves**	**33.71 ± 0.49^b^**	**46.93 ± 1.82^a^**	**34.88 ± 0.32^a^**	**43.84 ± 1.47^a^**	**33.92 ± 1.47^a^**	**35.31 ± 0.98^b^**
	**Fruits**	**61.01 ± 4.15^d^**	**93.87 ± 6.95^a^**	**74.41 ± 1.46b^c^**	**97.62 ± 4.35^a^**	**71.42 ± 1.54^c^**	**82.09 ± 4.52^b^**
**Total phenols** **(mg. g^-1^ FM)**		**13.76 ± 1.67^a^**	**15.81 ± 1.89^a^**	**40.58 ± 8.01^b^**	**63.38 ± 5.73 ^c^**	**42.32 ± 4.77^b^**	**35.05 ± 3.87^b^**

Control: no fertilizers; CF: chemical fertilizer, optimal dose of NPK (positive control); Bg22c: inoculation with the *Serratia* sp. isolate Bg22c; Bg32c: with *Pseudomonas *sp. isolate Bg32c; Bg15d: with *Pseudomonas *sp. isolate Bg15d; and Bg22c + Bg32c: with the bacterial consortium of isolates Bg22c and Bg32c. The values of each parameter labeled by different letters indicate significant differences assessed by Tukey’s test after performing a one-way ANOVA (P< 0.05).

#### Phosphorus analysis

**Figure 4:**
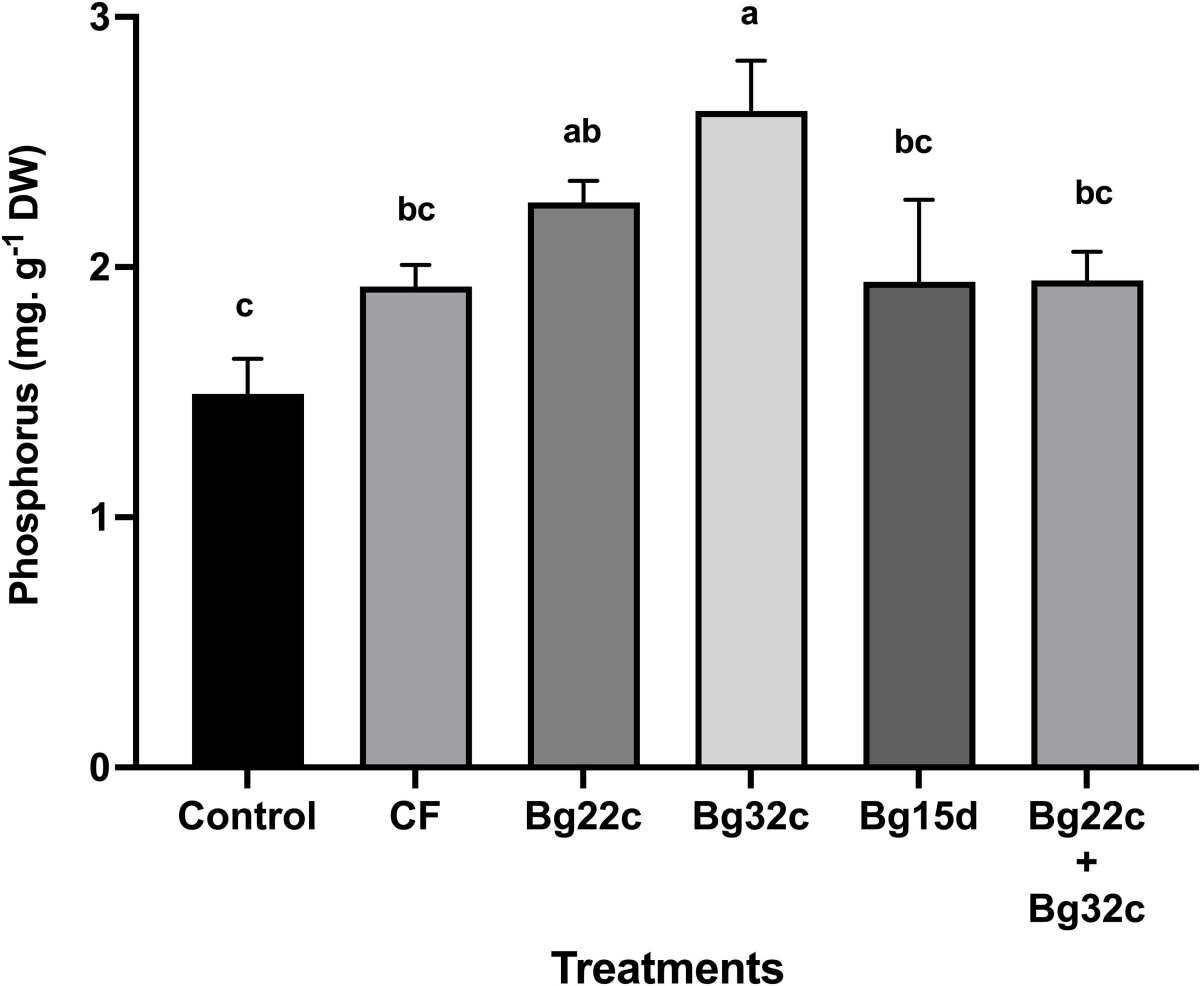
Effects of different phosphate solubilizing bacteria and chemical fertilizer (NPK) on the phosphorus content of cherry tomato. Control: absence of the tested biofertilizers, CF: chemical fertilizer (positive control): application of the optimal dose of NPK, Bg22c: inoculation with *Serratia* sp. isolate Bg22c, Bg32c: with *Pseudomonas* sp. isolate Bg32c, Bg15d: with *Pseudomonas* sp. isolate Bg15d, and Bg22c + Bg32c: with the bacterial consortium of isolates Bg22c and Bg32c. Different letters are significantly different (P<0.05) by Tukey test.

Plant phosphorus was enhanced by the application of phosphate solubilizing bacteria (**Figure 4**). Shoot phosphorus content increased in plants inoculated with Bg22c, Bg32c, Bg15d, and the combination by 51.3, 75.7, 30, and 30.3 respectively. Bg32c was recorded as the most effective inoculation.

### Principal component analysis

**Figure 5:**
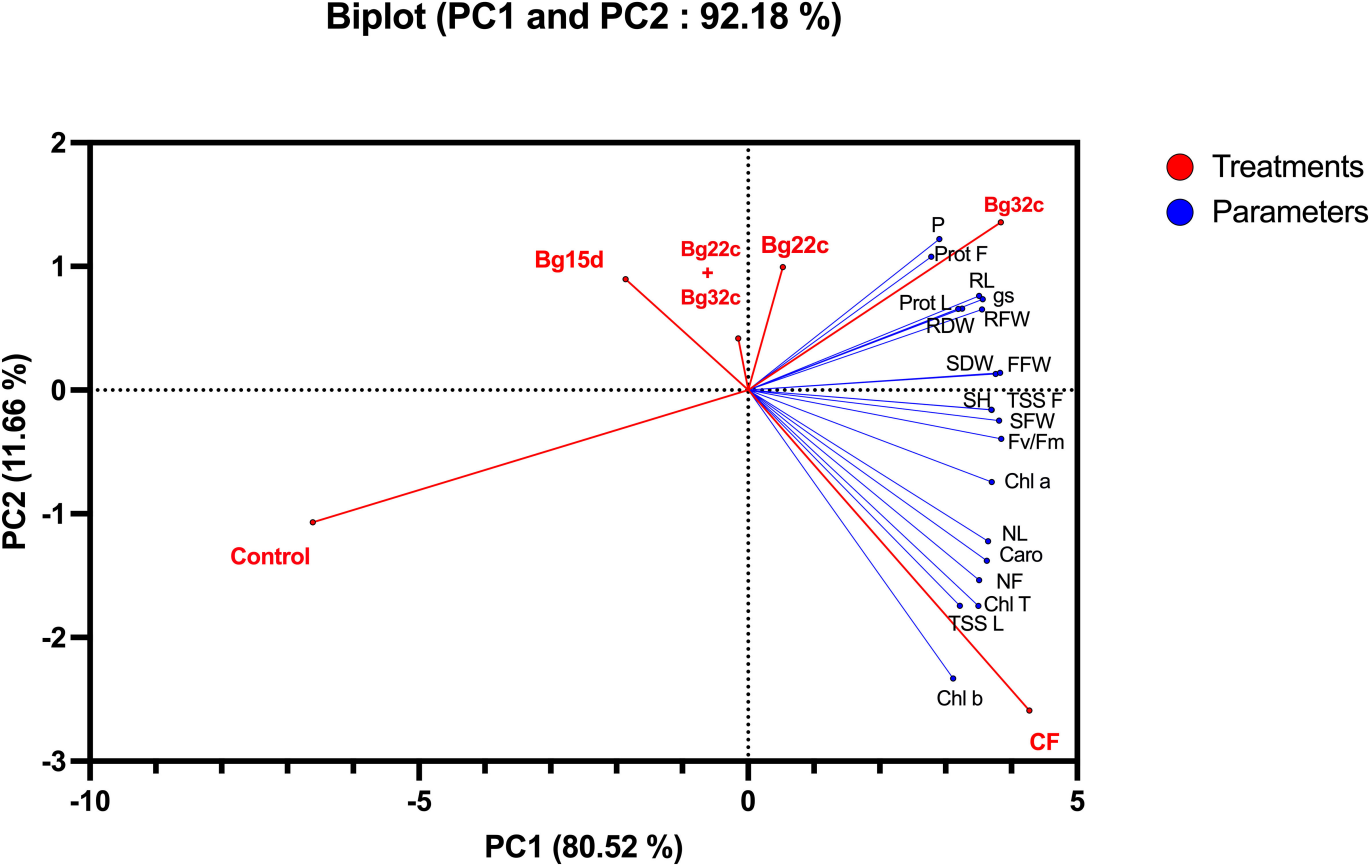
Principal component of cherry tomato plants subjected to different phosphate solubilizing bacteria and chemical fertilizer (NPK). The variables (agro-physiological, biochemical, and phosphorus) are represented in red. The treatments are given in blue. Control: absence of the tested biofertilizers; CF: chemical fertilizer (positive control): application of the optimal dose of NPK; Bg22c: inoculation with *Serratia* sp. isolate Bg22c; Bg32c: with *Pseudomonas* sp. isolate Bg32c; Bg15d: with *Pseudomonas* sp. isolate Bg15d; and Bg22c + Bg32c: with the bacterial consortium of isolates Bg22c and Bg32c. SH, shoot height; NL, number of leaves; RL, root length; SFW, shoot fresh weight; RFW, root fresh weight; SDW, shoot dry weight; RDW, root dry weight; FFW, fruit fresh weight; NF, number of fruits; gs, stomatal conductance; Fv/Fm, chlorophyll fluorescence; TSS L, total soluble sugar in leaves; TSS F, total soluble sugar in fruits; Prot L, protein content in leaves; Prot F, protein content in Fruit; Car, carotenoids; Chl a, chlorophyll a; Chl b, chlorophyll b; Chl t, total chlorophyll; p, phosphorus in leaves.

To assess the contributions of each parameter in control and phosphate solubilizing bacteria-treated tomato plants, we performed a principal component analysis using morphological, physiological, biochemical, and post-harvest mineral parameters collected from the plants. The principal component analysis shows that treatments (red) and variables (blue) were associated with two top PCs accounting for 92.18% of the total variation in traits under greenhouse conditions (**Figure 5**). PC1 explained 80.52% of the total variation and was strongly influenced by morphological and biochemical parameters, while PC2 accounted for 11.66% of the total variation and was strongly associated with physiological parameters related to the photosynthetic machinery. The PCA analysis showed a positive correlation between applied phosphate solubilizing bacteria and growth parameters, photosynthetic pigments and sugar, and protein content of leaves and fruits, which were positively correlated with each other. The analysis also confirmed the positive impact of the bacteria used, namely Bg22c and Bg32c, on these parameters. The biplot revealed the positive correlation between the applied bacteria Bg22c and Bg32c with the parameters of growth, physiology, biochemistry, and nutrition. In addition, PCA showed that all applied treatments were separated from their controls. Growth, physiological, biochemical, and mineral (phosphorus) parameters grouped the applied treatments into four main groups; the best treatments in terms of higher growth and efficient photosynthetic and biochemical parameters by treatments with strains Bg22c and Bg32c are on the right side of the first axis (PC1) (upper panel). In addition, the chemical fertilizer NPK-treated plants (right side of the first axis, lower panel) represent intermediate growth and better efficient photosynthetic system. In contrast, the control treatment without inoculation showed lower growth and accumulation of aboveground and root biomass.

### Identification of the bacterial isolates

**Figure 6:**
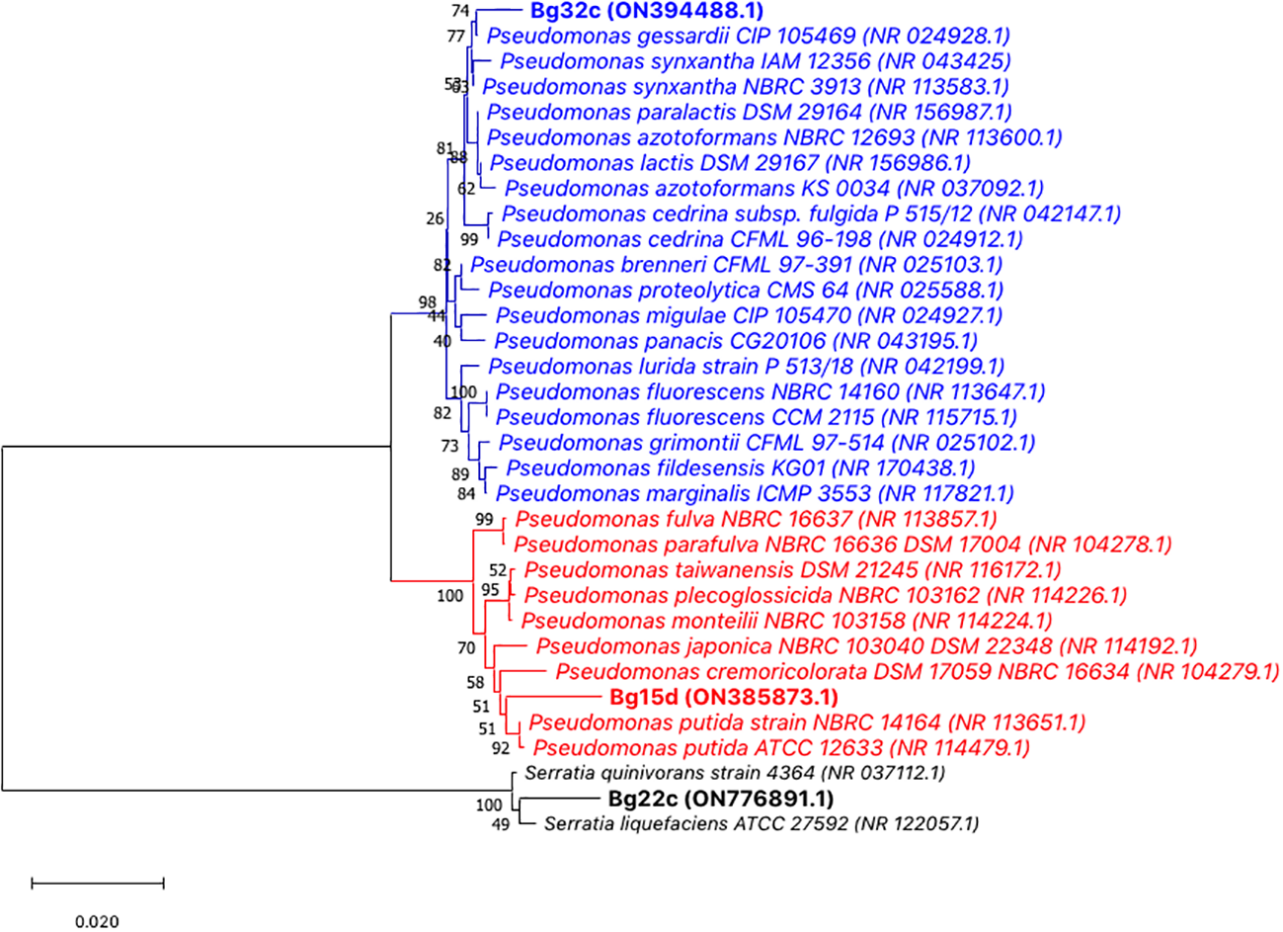
Consensus phylogenetic tree of the three bacterial strains and closest species based on almost complete 16S rRNA gene sequences (1500 bp) inferred using the Neighbor–Joining method (Saitou and Nei, 1987). The percentage of replicate trees in which the associated taxa clustered together in the bootstrap test (1000 replicates) are shown next to the branches (Felsenstein, 1985). The evolutionary distances were computed using the Maximum Composite Likelihood method (Tamura et al., 2004) and are in the units of the number of base substitutions per site. Bar represents nucleotide substitutions per site. The proportion of sites where at least one unambiguous base is present in at least one sequence for each descendent clade is shown next to each internal node in the tree. There were a total of 1579 positions in the final dataset. GenBank accession numbers of the strains are presented in brackets. Evolutionary analyses were conducted in MEGA 11 (Stecher et al., 2020; Tamura et al., 2021). Bg32c was close to *Pseudomonas gessardii* CIP 105469 (99.28% sequence identity) and *P. synxantha* NBRC3913 (99.21%. sequence identity). Bg15d shared low sequence similarities with *Pseudomonas putida* strains NBRC14164 and strain ATCC 12633 (98.35%) and other *Pseudomonas* species. Bg22c shared low sequence similarities with *Serratia quinivorans* 4364 (98.76%) and *S. liquefaciens* (98.54%).

The two isolates Bg22c and Bg32c were identified using classical phenotypic and molecular genotypic techniques. All isolates were Gram negative, unicellular rods with circular white to yellow colonies, and were oxidase positive. They were also positive for indole, Levan, and tween. No strains produced H_2_S and none could assimilate citrate. Furthermore, they failed to hydrolyze gelatin and starch. Based on 16S rRNA gene sequences, the molecular identification revealed that the two isolates, Bg32c and Bg15d, belong to species of the genus *Pseudomonas*, while Bg22c could belong to the genus *Serratia*. Phylogenetic tree constructed using MEGA 11 with neighbor-joining method supported by a 1000 bootstrap value showed that Bg32c shared high similarity with the type strains *Pseudomonas gessardii* (99.28% sequence identity) and *P. synxantha* (99,21% sequence identity). Bg32c, however, shared lower sequence identity, below the species delineation threshold, with other *Pseudomonas* species. It shares 98.99%, 98.91%, 98.78%, 98.63%, and 98.21% sequence identity, respectively with the type strains *P. paralactis*, *P. lactis*, *P. cedrina*, *P. azotoformans*, and *P. fluorescens*, and only 96.42%, 95.85%, and 95.70%, respectively with *P. fulva, P. plecoglossicida*, and *P. putida.* Strain Bg15d shares low sequence similarities, below the species threshold, with representatives of *Pseudomonas* species (*P. putida*: 98.35%, *P. taiwanensis*: 98.21%, *P. monteilii*: 98.07%, *P. plecoglossicida*: 98.14%, *P. japonica*: 97.99%*, P. cremoricolorata*: 97.85%, and *P. fluorescens*: 95.57%). Bg22c shared 98.76% sequence identity with *Serratia quinivorans* and 98,54% with *S. liquefaciens*. However, those values are below the species delineation threshold (**Figure 6**).

## Discussion

Tomatoes are one of the most economically important crops in the world. Today, tomato crop yield is limited by the low levels of phosphorus in the soil (Sanjuan-Delmás et al., 2020). Numerous studies have reported that bacteria belonging to the genera *Pseudomonas* (Cheng et al., 2012; Phour and Sindhu, 2020) and *Bacillus* (Misra and Chauhan, 2020) have plant growth-promotion abilities including production of ACC deaminase, phytohormones (auxins, cytokinins, and giberellins), and siderophores. In addition, bacteria solubilize phosphate (P) and exhibit antagonistic properties against phytopathogens (Bouizgarne, 2013). Among phosphate solubilizing bacteria, *Pseudomonas* are inhabitants of a wide range of environments and exhibit great metabolic diversity (Lugtenberg and Kamilova, 2009; Robin et al., 2008; Bouizgarne, 2013; Bouizgarne and Ait Ben Aoumar, 2014; Bouizgarne, 2022). Phosphorus in the soil solution exists as insoluble inorganic phosphorus and insoluble organic phosphorus. Most soils are P deficient and, despite the application of chemical fertilizers to the soil, plants are only able to use small amounts of phosphorus fertilizers (Bouizgarne et al., 2022). On the other hand, environmental issues related to the use of chemical phosphate fertilizers are a major challenge (Mäder et al., 2002). Therefore, research for ecofriendly alternatives is needed. Highly efficient phosphate solubilizing bacteria (PSB), applied either alone or in combination with chemical fertilizers able to significantly increase phosphorus content in the plant rhizosphere and crop yield, could represent an ecofriendly strategy (Hussain et al., 2021). Moroccan phosphate mines are an unexplored biotope and could represent an interesting source of well adapted phosphate solubilizing microorganisms. Therefore, the objective of the present study was to isolate Moroccan phosphate mine native fluorescent *Pseudomonas* with effective phosphate solubilization, investigating their *in vitro* PGP abilities, their potential as antagonists to tomato bacterial canker agent, *Clavibacter michiganensis* subsp. *michiganensis*, and their potential to promote tomato plant growth in greenhouses.

Our results revealed that two isolates, Bg22c and Bg32c, with *in vitro* abilities to solubilize phosphate, potash, and insoluble zinc sources were more efficient in the greenhouse experiment compared to the control and the non-solubilizer isolate Bg15g. Molecular identification revealed that Bg32c and Bg15d are close to *Pseudomonas* while Bg22c was close to *Serratia*. However, Bg22c and Bg15d are potential new species as they are separated from their closest neighbors according to pairwise distance calculated following 16S rRNA gene sequences. Indeed, pairwise similarities are below the threshold value of 98.8% for species delineation. This suggests that those strains are new species. Tomato plants inoculated with the isolate Bg32c resulted in a higher increase in growth parameters (shoot height, root length, number of leaves, number of fruits, shoot and root biomass, and fresh weight of fruits) compared to untreated plants, suggesting the positive role of these bacteria in growth promotion. Results showed that tomato treated with bacteria have significant effects on the shoot and root biomasses. It has been reported that phosphate solubilizing bacteria can promote plant growth through increased nitrogen uptake, synthesis of phytohormones, solubilization of inorganic and/or organic phosphates in the soil, and production of siderophores that chelate iron and make it available to the plant root (Habil-Addas et al., 2017; Azaroual et al., 2020; Bouizgarne, 2022). For instance, application of *Pseudomonas fluorescens* was reported to result in increases in shoot length, number of flowers, number of fruits, and total fruit weight in tomato plants treated by strains of *P. fluorescens* (Kandan, 2000; Mirza et al., 2001). Bacteria are able to modify the architecture of the root system through the production of phytohormones (Bouizgarne, 2013; Tahir et al., 2017). Recently, Chu et al. (2020) showed that *Pseudomonas* PS01 inhibited primary root elongation and triggered lateral root formation and root hair development. The increase in root architecture in inoculated plants may be due to the synthesis of the auxin phytohormone IAA, which play a major role in the development of the root system (Khare et al., 2018).

Phosphate solubilizing bacteria could represent a sustainable approach for managing phosphorus deficiency in agricultural soils (Sharma et al., 2013). In the present work, *Pseudomonas* and *Serratia* strains have been shown to improve tomato plant growth and development by providing essential nutrients such as phosphorus, zinc, and potassium. In addition, the selected bacteria produce auxin (IAA) that can promote and improve root growth and architecture (Gamalero et al., 2004). Solubilization was accompanied by a drop in pH suggesting that it involves acidification of the medium and organic acids as the main solubilizing mechanism, as confirmed by High Pressure Liquid Chromatography (HPLC). Organic acid production was shown to be involved in phosphate solubilization; the two strains Bg22c and Bg32c produced gluconic and oxalic acids as major organic acids. Organic acid production is the principal mechanism for solubilization of soil P (Chen et al., 2006; Khan et al., 2009; Bouizgarne, 2022). After nitrogen and phosphorus, potassium (K) is the third most important plant nutrient and has a key role in the growth, metabolism, and development of plants. It is involved in increasing plant resistance to biotic and abiotic stresses and is required to activate most enzymes involved in plant processes (Ahmad et al., 2016). It was also reported that the major mechanism of K solubilization is production of organic and inorganic acids (Jain et al., 2022). As for phosphorus, most K is unavailable for plant uptake, so potassium solubilizing bacteria can be used to solubilize potassium-insoluble minerals such as biotite, feldspat, muscovite, vermiculite, smectite, orthoclase, and mica to soluble forms, which are then available for plant uptake (Ahmad et al., 2016). Species from bacterial genera, including *Bacillus*, *Paenibacillus*, *Pseudomonas*, and *Burkholderia*, have been reported to possess K solubilization ability (Ahmad et al., 2016). Zinc is also among the essential micronutrients for plant growth and inorganic zinc in soil is an unsuitable form for plant assimilation. Zinc plays an essential role in the biosynthesis of IAA through the formation of its precursor, tryptophan. In addition, Zinc is involved in many other physiological functions: cell division, elongation, fruit development, control of gene expression, stabilizing RNA and DNA structure, and maintaining the activity of RNA degrading enzymes (Brown et al., 1993). Several studies reported an increase in plant biomass with the application of plant growth-promoting bacteria, especially due to increased mineral nutrition and nutrient uptake resulting in increased photosynthesis (Stefan et al., 2013; Kour et al., 2020). In tomato, bacteria with solubilizing abilities showed growth enhancement (Sharon et al., 2016; Zhang et al., 2017; Guerrieri et al., 2020). Our results suggest that the selected bacteria could be involved in transforming inorganic, insoluble potash and zinc forms into forms biologically available for plants. Treatment with Bg32c resulted in a high number and weight of fruits per plant probably due to P, K, and Zn solubilization in addition to the production of auxin. Indeed, it was found that the plant growth hormones produced by the bacterial strains are involved in increasing the efficiency of nutrient utilization in the inoculated plants. The applied phosphate solubilizing bacteria showed significant positive effects on plant physiological parameters. An increase in chlorophyll content (Chl a, Chl b, and Chl T), carotenoid content, stomatal conductance, and increase in quantum yield was recorded in Bg32c treated plants. The increase in stomatal conductance and quantum yield, as well as the increase in carotenoid content of inoculated plants, could be due to the increase in chlorophyll content. Studies reported by Seema et al. (2018) showed the positive effect of applied bacteria to stomatal conductance, quantum yield, and chlorophyll content. Bacteria have the ability to contribute to the enhancement of N, Ca, Mg, and K acquisition in plants (Abd-Allah et al., 2018). Increased photosynthesis, stomatal conductance (maximum stomatal opening), and quantum yield may result from increased chlorophyll content and maximum CO_2_ assimilation (Misratia et al., 2013). The higher total sugars and proteins recorded in plants inoculated with the phosphate solubilizing bacteria used, especially Bg32c, may be due to increased leaf biomass and increased photosynthesis, resulting in increased carbohydrate accumulation and efficient distribution of photosynthates. In addition, this increase in total soluble sugars and protein content may be due to increased availability of mineral nutrients. The isolate application of adapted bacterial strains from tomato rhizosphere was found to be an effective strategy to improve tomato growth and physiology. In this work, we searched for *Pseudomonas* adapted to a phosphate mine environment as a promising strategy. In our previous works, we showed that isolation of *Bacillus* strains and Mycorrhizae from this environment led to efficient growth in wheat (Azaroual et al., 2020; Hazzoumi et al., 2022). Under semi-controlled conditions, inoculation with phosphate solubilizing bacteria Bg32c, Bg22c, and the consortium (Bg22c + Bg32c), resulted in improved growth and physiological and biochemical parameters of cherry tomato compared to the negative control. Strain Bg32c bacteria proved to be the most efficient. All measured parameters were significantly higher with inoculation compared to the untreated (control) treatment. Moreover, this bacterium showed no significant difference from conventional chemical fertilizer (NPK) for the parameters evaluated in this study. Altogether, the two strains Bg32c and Bg22c are interesting, and results should encourage their use as good biofertilizers candidates either individually or in combination. The two isolates, Bg22c and Bg15d, could inhibit the growth of the causal agent of tomato bacterial canker, *Clavibacter michiganensis* subsp. *michiganensis in vitro*. All the isolates were also able to trigger phenolic compounds synthesis, which suggest their potential usefulness as inducers of systemic resistance mechanisms in tomato that could enhance plant defense against pathogens.

Thus, our experimental results indicate that the bacteria close to *Pseudomonas* used in this study, especially Bg32c, could be valuable and sustainable biological tools as biofertilizer for improving growth and yield of cherry tomato, as well as biopesticides against *Clavibacter michiganensis* subsp. *michiganensis*, the most devastating disease of marketable tomato in greenhouses. All results suggest the application of these bacteria as a sustainable biological alternative for chemicals. In addition, Moroccan phosphate mines could be a source of potential new *Pseudomonas* species for which new names will be given. Experiments aiming to protect tomato seedlings from bacterial canker disease are in progress.

## Data availability statement

The datasets presented in this study can be found in online repositories. The names of the repository/repositories and accession number(s) can be found below: https://www.ncbi.nlm.nih.gov/genbank/, ON394488 https://www.ncbi.nlm.nih.gov/genbank/, ON776891 https://www.ncbi.nlm.nih.gov/genbank/, ON385873.

## Author contributions

BB and MA contributed to the conception and design of the study. MB and BB contributed to the isolation, screening, solubilization measurements, and identification of the bacteria, SAEM, AA and GA contributed to experiment aiming to check for activities of solubilization and antagonistic activities *in vitro* after conservation, BB, BA and MA contributed to experiments in greenhouse in controlled conditions and measurements, BB wrote/corrected the manuscript, MB, BA and BaB contributed to statistic analyses. All authors contributed to the article and approved the submitted version.

## Supplementary material

Supplementary Figure 1 Chromatograms of the organic acids in the supernatant of the bacterial isolates Bg22c **(A)** and Bg32c **(B)** cultured in NBRIP containing TCP as P source.

Supplementary Figure 2 Effects of application of phosphate solubilizing *Serratia* sp. isolate Bg22c and *Pseudomonas* sp. isolate Bg32c compared to non solubilizing isolate, *Pseudomonas* sp. Bg15d, negative control, and a chemical fertilizer, CF (positive control) on cherry tomato growth.

## References

[B1] Abd-Allah E. F. Alqarawi A. A. Hashem A. Radhakrishnan R. Al-Huqail A. A. Al-Otibi F. O. N.. (2018). Endophytic bacterium *Bacillus subtilis* (BERA 71) improves salt tolerance in chickpea plants by regulating the plant defense mechanisms. J. Plant Interact. 13, 37–44. doi: 10.1080/17429145.2017.1414321

[B2] Adesemoye A. O. Torbert H. A. Kloepper J. W. (2009). Plant growth-promoting rhizobacteria allow reduced application rates of chemical fertilizers. Microb. Ecol. 58, 921–929. doi: 10.1007/s00248-009-9531-y 19466478

[B3] Ahmad M. Nadeem S. M. Naveed M. Zahir Z. A. (2016). “Potassium-solubilizing bacteria and their application in agriculture”. in Potassium solubilizing microorganisms for sustainable agriculture, eds. Meena V. Maurya B. Verma J. Meena R., (Springer, New Delhi), doi: 10.1007/978-81-322-2776-2_21

[B4] Arnon D. (1949). Copper enzymes in isolated chloroplasts. polyphenoloxidase in *Beta vulgaris* . Plant Physiol. 24, 1–15. doi: 10.1104/pp.24.1.1 16654194 PMC437905

[B5] Azaroual S. E. Hazzoumi Z. El Mernissi N. Aasfar A. Meftah K. I. Bouizgarne B. (2020). Role of inorganic phosphate solubilizing *Bacilli* isolated from Moroccan phosphate rock mine and rhizosphere soils in wheat (*Triticum aestivum* l) phosphorus uptake. Curr. Microbiol. 77, 2391–2404. doi: 10.1007/s00284-020-02046-8 32468184

[B6] Berrada I. Willems A. De Vos P. El Fahime E. M. Swings J. Bendaou N.. (2012). Diversity of culturable moderately halophilic and halotolerant bacteria in a marsh and two salterns a protected ecosystem of Lower Loukkos (Morocco). Afr. J. Microbiol. Res. 6, 2419–2434. doi: 10.5897/AJMR-11-1490

[B7] Bouizgarne B. (2013). “Bacteria for plant growth promotion and disease management,” in Bacteria in agrobiology: Disease management. Ed. Maheshwari D. K. (Berlin, Heidelberg: Springer). doi: 10.1007/978-3-642-33639-3_2

[B8] Bouizgarne B. (2022). “Phosphate-solubilizing actinomycetes as biofertilizers and biopesticides: bioformulations for sustainable agriculture,” in Microbial BioTechnology for sustainable agriculture, volume 1. microorganisms for sustainability, vol. vol 33 . Eds. Arora N. K. Bouizgarne B. (Singapore: Springer). doi: 10.1007/978-981-16-4843-4_13

[B9] Bouizgarne B. Ait Ben Aoumar A. (2014). “Diversity of plant associated actinobacteria,” in Bacterial diversity in sustainable agriculture. Ed. Maheshwari D. K. (Switzerland: Springer), 41–99.

[B10] Bouizgarne B. El Hadrami I. Ouhdouch Y. (2006). Novel production of isochainin by a strain of *Streptomyces* sp. isolated from rhizosphere soil of the indigenous Moroccan plant *Argania spinosa* L. W. J. Microbiol. Biotechnol. 22, 423–429. doi: 10.1007/s11274-005-9051-y

[B11] Bradford M. M. (1976). A rapid and sensitive method for the quantitation microgram quantities of protein utilizing the principle of protein-dye binding. Crop J. 5, 407–415. doi: 10.1016/j.cj.2017.04.003 942051

[B12] Bric J. M. Bostock R. M. Silverstone S. E. (1991). Rapid *in situ* assay for indoleacetic acid production by bacteria immobilized on a nitrocellulose membrane. Appl. Environ. Microbiol. 57, 535–538. doi: 10.1128/aem.57.2.535-538.1991 16348419 PMC182744

[B13] Brown P. H. Cakmak I. Zhang Q. (1993). “Form and function of Zinc plants”, in Zinc in soils and plants, ed. Robson A. D. (Kluwer Acadamic Publishers, Dordrecht), 93–106. doi: 10.1007/978-94-011-0878-2_7

[B14] Bunt J. S. Rovira A. D. (1955). Microbiological studies of some subantartic soils. J. Soil Sci. 6, 119−128. doi: 10.1111/j.1365-2389.1955.tb00836.x

[B15] Chen Y. P. Rekha P. D. Arun A. B. Shen F. T. Lai W.-A. Young C. C. (2006). Phosphate solubilizing bacteria from subtropical soil and their tricalcium phosphate solubilizing abilities. Appl. Soil Ecol. 1, 33–41. doi: 10.1016/j.apsoil.2005.12.002

[B16] Cheng Z. Woody O. Z. McConkey B. J. Glick B. R. (2012). Combined effects of the plant growth-promoting bacterium *Pseudomonas putida* UW4 and salinity stress on the *Brassica napus* proteome. Appl. Soil Ecol. 61, 255–263. doi: 10.1016/j.apsoil.2011.10.006

[B17] Chu T. N. Bui L. V. Hoang M. T. T. (2020). *Pseudomonas* PS01 isolated from maize rhizosphere alters root system architecture and promotes plant growth. Microorganisms 8, 471. doi: 10.3390/microorganisms8040471 32224990 PMC7232511

[B18] Chung H. Park M. Madhaiyan M. Seshadri S. Song J. Cho H.. (2005). Isolation and characterization of phosphate solubilizing bacteria from the rhizosphere of crop plants of Korea. Soil Biol. Biochem. 37, 1970–1974. doi: 10.1016/j.soilbio.2005.02.025

[B19] de Sousa S. M. de Oliveira C. A. Andrade D. L. de Carvalho C. G. Ribeiro V. P. Pastina. (2021). Tropical *Bacillus* strains inoculation enhances maize root surface area, dry weight, nutrient uptake and grain yield. J. Plant Growth Regul. 40, 867–877. doi: 10.1007/s00344-020-10146-9

[B20] Dubois M. Gilles K. A. Hamilton J. K. Rebers P. A. Smith F. (1956). Colorimetric method for determination of sugars and related substances. Anal. Chem. 28, 350–356. doi: 10.1021/ac60111a017

[B21] Etesami H. Emami S. Alikhani H. A. (2017). Potassium solubilizing bacteria (KSB): Mechanisms, promotion of plant growth, and future prospects–a review. J. Soil Sci. Plant Nutr. 17, 897–911. doi: 10.4067/S0718-95162017000400005

[B22] Felsenstein J. (1985). Confidence limits on phylogenies: an approach using the bootstrap. Evol. 39, 783–791. doi: 10.2307/2408678 28561359

[B23] Fiske C. Subbarow Y. (1925). The colorimetric determination of phophorus. Biol. Chem. 66, 375–400. doi: 10.1016/S0021-9258(18)84756-1

[B24] Gamalero E. Trotta A. Massa N. Copetta A. Martinotti M. G. Berta G. (2004). Impact of two fluorescent pseudomonads and an arbuscular mycorrhizal fungus on tomato plant growth, root architecture and P acquisition. Mycorrhiza 14, 185–192. doi: 10.1007/s00572-003-0256-3 15197635

[B25] Gravel V. Antoun H. Tweddell R. J. (2007). Growth stimulation and fruit yield improvement of greenhouse tomato plants by inoculation with *Pseudomonas putida* or *trichoderma atroviride*: Possible role of indole acetic acid (IAA). Soil Biol. Biochem. 8, 1968–1977. doi: 10.1016/j.soilbio.2007.02.015

[B26] Guerrieri M. C. Fanfoni E. Fiorini A. Trevisan M. Puglisi E. (2020). Isolation and screening of extracellular PGPR from the rhizosphere of tomato plants after long-term reduced tillage and cover crops. Plants. 9, 668. doi: 10.3390/plants9050668 32466288 PMC7285081

[B27] Gulati A. Sharma N. Vyas P. Sood S. Rahi P. Pathania V.. (2010). Organic acid production and plant growth promotion as a function of phosphate solubilization by *Acinetobacter rhizosphaerae* strain BIHB 723 isolated from the cold deserts of the trans-Himalayas. Arch. Microbiol. 192, 975–983. doi: 10.1007/s00203-010-0615-3 20821196

[B28] Habil-Addas F. El, Aarab S. Rfaki A. Laglaoui A. Bakkali M. Arakrak A. (2017). Screening of phosphate solubilizing bacterial isolates for improving growth of wheat. Eur. J. Biotechnol. Biosci. 5, 7–11.

[B29] Harley P. C. Loreto F. Marco G. Sharkey T. D. (1992). Theoretical considerations when estimating the mesophyll conductance to CO_2_ flux by analysis of the response of photosynthesis to CO_2_ . Plant Physiol. 98, 1429–1436. doi: 10.1104/pp.98.4.1429 16668811 PMC1080368

[B30] Hazzoumi Z. Azaroual S. E. El Mernissi N. Zaroual Y. Duponnois R. Bouizgarne B.. (2022). Effect of arbuscular mycorrhizal fungi isolated from rock phosphate mine and agricultural soil on the improvement of wheat plant growth. Front. Microbiol. 13. doi: 10.3389/fmicb.2022.881442 PMC9174796 35694314

[B31] Hussain S. Sharif M. Ahmad W. (2021). Selection of efficient phosphorus solubilizing bacteria strains and mycorrhizea for enhanced cereal growth, root microbe status and N and P uptake in alkaline calcareous soil. Soil Sci. Plant Nutr. 67, 1–10. doi: 10.1080/00380768.2021.1904793

[B32] Jain D. Saheewala H. Sanadhaya S. Joshi A. Bhojiya A. A. Verma A. K.. (2022). “Potassium solubilizing microorganisms as soil health engineers: An insight into molecular mechanism,” in Rhizosphere engineering. Eds. Dubey R. C. Kumar P. (Academic Press), 199–214.

[B33] Kandan A. (2000). Induction of systemic resistance against tomato spotted wilt virus (TSWV) in tomato by fluorescent *Pseudomonas* strains (Tamil Nadu: Agriculture University Combatore, India).

[B34] Khan A. A. Jilani G. Akhtar M. S. Naqvi S. M. S. Rasheed M. (2009). Phosphorus solubilizing bacteria: occurrence, mechanisms and their role in crop production. J. Agric. Biol. Sc. 1, 48–58.

[B35] Khare E. Mishra J. Arora N. K. (2018). Multifaceted interactions between endophytes and plant: Developments and prospects. Front. Microbiol. 9. doi: 10.3389/fmicb.2018.02732 PMC6249440 30498482

[B36] Kour D. Rana K. L. Yadav A. N. Yadav N. Kumar M. Kumar V.. (2020). Microbial biofertilizers: Bioresources and eco-friendly technologies for agricultural and environmental sustainability. *Biocatal* . Agric. Biotechnol. 23, 101487. doi: 10.1016/j.bcab.2019.101487

[B37] Lugtenberg B. Kamilova F. (2009). Plant-growth-promoting rhizobacteria. Annu. Rev. Microbiol. 63, 541–556. doi: 10.1146/annurev.micro.62.081307.162918 19575558

[B38] Macheix J. J. Fleuriet A. Billot J. (1990). Fruit phenolics. Boca Raton, FL: CRC Press, pp. 1–126, ISBN 9781315893075, doi: 10.1201/9781351072175

[B39] Mäder P. Fließbach A. Dubois D. Gunst L. Fried P. Niggli U. (2002). Soil fertility and biodiversity in organic farming. Science. 296, 1694–1697. doi: 10.1126/science.1071148 12040197

[B40] Marra L. M. de Oliveira-Longatti S. M. Soares C. R. de Lima J. M. Olivares F. L. Moreira F. M. (2015). Initial pH of medium affects organic acids production but do not affect phosphate solubilization. Braz. J. Microbiol. 46, 367–375. doi: 10.1590/S1517-838246246220131102 26273251 PMC4507528

[B41] Mirza M. S. Ahmad W. Latif F. Haurat J. Bally R. Normand P.. (2001). Isolation, partial characterization, and the effect of plant growth-promoting bacteria (PGPB) on micro-propagated sugarcane *in vitro* . Plant Soil. 237, 47–54. doi: 10.1023/A:1013388619231

[B42] Misra S. Chauhan P. S. (2020). ACC deaminase-producing rhizosphere competent *Bacillus* spp. mitigate salt stress and promote *Zea mays* growth by modulating ethylene metabolism. 3 Biotech. 10, 1–14. doi: 10.1007/s13205-020-2104-y 32117680 PMC7024073

[B43] Misratia K. M. Ismail M. R. Hakim A. Musa M. H. Puteh A. (2013). Effect of salinity and alleviating role of gibberellic acid (GA3) for improving the morphological, physiological and yield traits of rice varieties. Aust. J. Crop Sci. 7, 1682–1692.

[B44] Nautiyal C. S. (1999). An efficient microbiological growth médium for screening phosphate solubilizing microorganisms. FEMS Microbiol. Lett. 170, 265–270. doi: 10.1111/j.1574-6968.1999.tb13383.x 9919677

[B45] Olsen S. Sommers L. (1982). Phosphorus, in methods of soil analysis: Part 2. chemical and microbiological properties. ed. Page A. L. Am. Soc. Agronomy. 403–430. doi: 10.2134/agronmonogr9.2.2ed

[B46] Phour M. Sindhu S. S. (2020). Amelioration of salinity stress and growth stimulation of mustard (*Brassica juncea* L.) by salt-tolerant *Pseudomonas* species. Appl. Soil Ecol. 149, 103518. doi: 10.1016/j.apsoil.2020.103518

[B47] Plaxton W. C. Tran H. T. (2011). Metabolic adaptations of phosphate-starved plants. Plant Physiol. 156, 1006–1015. doi: 10.1104/pp.111.175281 21562330 PMC3135920

[B48] Rajan S. S. S. Watkinson J. H. Sinclair A. G. (1996). Phosphate rocks for direct application to soils. Adv. Agron. 57, 77–159. doi: 10.1016/S0065-2113(08)60923-2

[B49] Robin A. Vansuyt G. Hinsinger P. Meyer J. M. Briat J. F. Lemanceau P. (2008). “Iron dynamics in the rhizosphere: Consequences for plant health and nutrition,” in Advances in agronomy, vol. 99 . Ed. Sparks D. L. (London, UK: Academic Press), 183–225.

[B50] Saitou N. Nei M. (1987). The neighbor-joining method: a new method for reconstructing phylogenetic trees. Mol. Biol. Evol. 4, 406–425. doi: 10.1093/oxfordjournals.molbev.a040454 3447015

[B51] Sanjuan-Delmás D. Josa A. Muñoz P. Gassó S. Rieradevall J. Gabarrell X. (2020). Applying nutrient dynamics to adjust the nutrient-water balance in hydroponic crops. a case study with open hydroponic tomato crops from Barcelona. Sci. Hortic. 261, 108908. doi: 10.1016/j.scienta.2019.108908

[B52] Seema K. Mehta K. Singh N. (2018). Studies on the effect of plant growth promoting rhizobacteria (PGPR) on growth, physiological parameters, yield and fruit quality of strawberry cv. chandler. J. pharmacogn. phytochem. 7, 283–387.

[B53] Sharma S. B. Sayyed R. Z. Trivedi M. H. Gobi T. A. (2013). Phosphate solubilizing microbes: sustainable approach for managing phosphorus deficiency in agricultural soils. Springer plus 2, 587–600. doi: 10.1186/2193-1801-2-587 25674415 PMC4320215

[B54] Sharon J. A. Hathwaik L. T. Glenn G. M. Imam S. H. Lee C. C. (2016). Isolation of efficient phosphate solubilizing bacteria capable of enhancing tomato plant growth. J. Soil Sci. Plant Nutr. 16, 525–536. doi: 10.4067/S0718-95162016005000043

[B55] Stecher G. Tamura K. Kumar S. (2020). Molecular evolutionary genetics analysis (MEGA) for macOS. Mol. Biol. Evol. 37, 1237–1239. doi: 10.1093/molbev/msz312 31904846 PMC7086165

[B56] Stefan M. Munteanu N. Stoleru V. Mihasan M. Hritcu L. (2013). Seed inoculation with plant growth promoting rhizobacteria enhances photosynthesis and yield of runner bean (*Phaseolus coccineus* L.). Sci. Hortic. 151, 22–29. doi: 10.1016/j.scienta.2012.12.006

[B57] Strasser B. J. Strasser R. J. (1995). “Measuring fast fluorescence transients to address environmental questions: The JIP-test,” in Photosynthesis: from light to biosphere, vol. 5 . Ed. Mathis P. (Dordrecht: KAP Press), 977–980. doi: 10.1007/978-94-009-0173-5_1142

[B58] Tahir H. A. S. Gu Q. Wu H. Raza W. Hanif A. Wu L.. (2017). Plant growth promotion by volatile organic compounds produced by *Bacillus subtilis* SYST2. Front. Microbiol. 8. doi: 10.3389/fmicb.2017.00171 PMC5293759 28223976

[B59] Tahir M. Khalid U. Ijaz M. Shah G. M. Naeem M. A. Shahid M.. (2018). Combined application of bio-organic phosphate and phosphorus solubilizing bacteria (*Bacillus* strain MWT 14) improve the performance of bread wheat with low fertilizer input under an arid climate. Braz. J. Microbiol. 49, 15–24. doi: 10.1016/j.bjm.2017.11.005 29728340 PMC6328723

[B60] Tamura K. Nei M. Kumar S. (2004). Prospects for inferring very large Phylogenies by using the Neighbor-Joining method. Proc. Natl. Acad. Sci. U.S.A. 101, 11030–11035. doi: 10.1073/pnas.0404206101 15258291 PMC491989

[B61] Tamura K. Stecher G. Kumar S. (2021). MEGA11: Molecular Evolutionary Genetics Analysis, Version 11. Mol. Biol. Evol. 38, 3022–3027. doi: 10.1093/molbev/msab120 33892491 PMC8233496

[B62] Wakelin S. A. Warren R. A. Harvey P. R. Ryder M. H. (2004). Phosphate solubilization by *Penicillium* spp. closely associated with wheat roots. Biol. Fert. Soils 40, 36–43. doi: 10.1007/s00374-004-0750-6

[B63] Wei Y. Zhao Y. Shi M. Cao Z. Lu Q. Yang T.. (2018). Effect of organic acids production and bacterial community on the possible mechanism of phosphorus solubilization during composting with enriched phosphate-solubilizing bacteria inoculation. Bioresour. Technol. 247, 190–199. doi: 10.1016/j.biortech.2017.09.092 28950126

[B64] Zhang J. Wang P. Fang L. Zhang Q. A. Yan C. Chen J. (2017). Isolation and characterization of phosphate-solubilizing bacteria from mushroom residues and their effect on tomato plant growth promotion. Polish J. Microbiol. 66, 57–65. doi: 10.5604/17331331.1234993 29359698

[B65] Zhu H. J. Sun L. F. Zhang Y. F. Zhang X. L. Qiao J. J. (2012). Conversion of spent mushroom substrate to biofertilizer using a stress-tolerant phosphate-solubilizing *Pichia farinose* FL7. Bioresour. Technol. 11, 410–416. doi: 10.1016/j.biortech.2012.02.042 22386630

