# Supplementary figures and images for: Phosphate and potash solubilizing bacteria from Moroccan phosphate mine showing antagonism to bacterial canker agent and inducing effective tomato growth promotion
Source: Front Plant Sci. 2023 Mar 8;14:970382. doi: 10.3389/fpls.2023.970382 (PMC10030999; doi:10.3389/fpls.2023.970382)

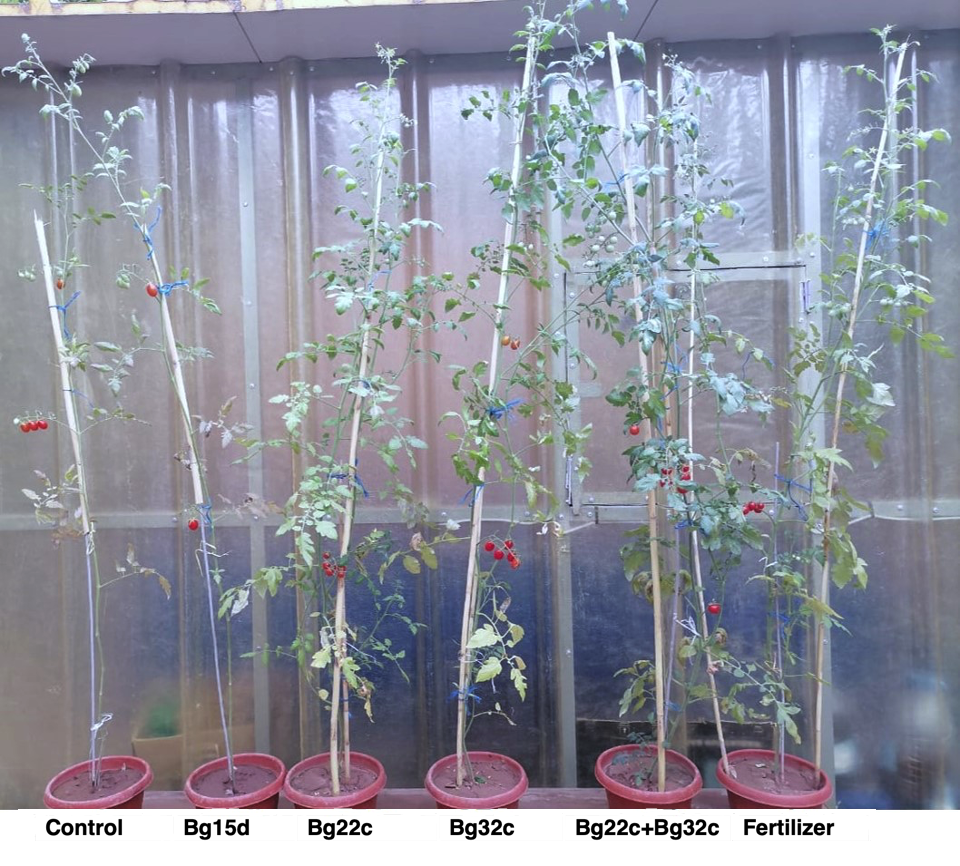

Supplement: Supplementary Figure 2 — Effects of application of phosphate solubilizing Serratia sp. isolate Bg22c and Pseudomonas sp. isolate Bg32c compared to non solubilizing isolate, Pseudomonas sp. Bg15d, negative control, and a chemical fertilizer, CF (positive control) on cherry tomato growth. [file Image_2.tif]
